# Medicine Shortages: From Assumption to Evidence to Action - A Proposal for Using the FMD Data Repositories for Shortages Monitoring

**DOI:** 10.3389/fmed.2021.579822

**Published:** 2021-02-03

**Authors:** François Bouvy, Mihai Rotaru

**Affiliations:** European Federation of Pharmaceutical Industries and Associations, Brussels, Belgium

**Keywords:** shortages, falsified medicines directive, parallel trade, quotas, serialization

## Abstract

Medicine shortages may negatively impact patient care and the patient experience. Shortages should be a priority of industry, supply chain stakeholders and national competent authorities, and deserve more than empathy or “lip-service” but serious engagement and action. Under the overarching principle that without correct measurements in place there cannot be any improvement in overall performance (1), stakeholders in the pharmaceutical supply chain, notably pharmaceutical manufacturers have made the call for all relevant sources of information to be used in order to provide additional intelligence about the root causes and drivers of shortages, including the identification of bottlenecks in the supply chain. This paper outlines a proposal for using the data stored in the interoperable network of national repositories being set up in the context of the Falsified Medicines Directive (Directive 2011/62/EU) and its Delegated Regulation 2016/161/EU on safety features for providing additional intelligence in monitoring shortages. The paper analyses the potential feasibility and readiness of using this data for monitoring shortages as well as the strengths and weaknesses of the approach. We explore also what are some of the other complementary data sources that could be analyzed in conjunction with the data in the repository system to sharpen the overall analysis. Lastly, we provide a theoretical but concrete use case for using the abovementioned data for better informing decisions to prevent and mitigate shortages. In doing so, we explain the interlink between patient needs at country level, demand from economic actors, intra-EU parallel trade and manufacturer-imposed supply quotas and propose a mechanism for collaboration between national competent authorities and supply chain stakeholders for early detection and action to prevent medicine shortages from occurring.

## Introduction

Medicine shortages may negatively impact patient care and the patient experience. Over the recent past all of us have been confronted with specific examples or stories of patients who cannot get access to their treatment and these examples are difficult to bear when shortages relate to medication for life-threatening diseases or involving children and where there are no therapeutic alternatives. For these reasons medicine shortages should be a priority of industry, supply chain stakeholders and national competent authorities, and deserve more than empathy or “lip-service” but serious engagement and action. The causes of unavailability of medicines in EU Member State markets are broadly 3-fold: (i) products not being authorized; (ii) products being authorized but not marketed; (iii) products being authorized and marketed but unavailable due to shortages. This article focuses on the last of these. Unequal availability and patient access to centrally approved medicines within the EU requires a separate in-depth discussion.^1^ Shortages are defined in various ways by different competent authorities (including EMA) and stakeholders. In line with manufacturers' public service obligation defined by Article 81 of Directive 2001/83/EU industry stakeholders have agreed to define a shortage of a medicinal product for human use as arising in the situation “when supply does not meet patient need at a national level for a period of more than two weeks.”^2^

Over the last number of years there have been growing reports^3^ regarding the increased prevalence of medicine shortages as well as their proliferation across EU Member States, therapy areas and number of patients impacted. Together with this growth in the number of shortages reported we observe also a heterogeneous landscape of definitions, data sources and methodologies for designating a medicine shortage, depending on countries, depending on the timeframe considered, the therapeutic area, the scope or the criticality of the medicine considered.^4^

Any work aimed at better understanding the causes of shortage in the supply chain and aspiring to propose meaningful solutions should start from the premises of the most correct measurement of the phenomena. Supply chains nowadays (including pharmaceuticals) rely on increasingly global and digitalized networks (1). This evolution has driven increased attention in the last 40 years, both by the industry and by academic research, on defining and developing the right performance measures and metrics (2) and equips us with a multitude of empirically tested supply chain measures and systems (1) under the overarching principle that without correct measurements in place there cannot be any improvement in overall performance (3). As such, there is a need to enrich the current count of individual shortage events with a more comprehensive set of supply chain performance measures.

This article outlines a proposal for using the European Medicines Verification System, set up in the context of the Falsified Medicines Directive, in order to provide additional intelligence in monitoring shortages. The article is structured into three parts: Part 2 details the various data contained in the EVMS, explains how the system works in practice, introduces the future connection with the EMA IDMP database, and explains the data access and ownership rules in the system. Part 3 introduces the main proposal of the paper: the use of such data for monitoring shortages. Here we introduce the concept of Net Stock Level at national level and suggest a formula for its calculation by National Competent Authorities and we also outline the current limitations with this approach. Part 4 contains a proposal for collaboration between National Competent Authorities and manufacturers in order to proactively monitor country stock levels via a “dashboard” type tool and proposes a pilot initiative to be set-up to gather real-life experience.

The use of the data repository systems for monitoring shortages is currently a theoretical proposal which, to our knowledge, has not yet been put in practice in any Member State. The aim of this article is to kick-start a debate into the potential suitability for using the system for such a purpose, build awareness and encourage supply chain stakeholders and National Competent Authorities to further explore this proposal, potentially via a pilot initiative to gather real-life experience.

## The European Medicines Verification System (EMVS)

The EU Falsified Medicines Directive and its Delegated Regulation (4) provides for the establishment of interoperable repositories or database systems (also called EMVS) containing unique identifiers (i.e., product code, serial number, batch number, expiry date, and where applicable, national reimbursement number) for prescription medicines. According to the Delegated Regulation the repositories system shall be set up and managed by a non-profit legal entity or entities established in the EU by manufacturers and marketing authorization holders (MAHs) of medicines bearing the safety features, in consultation with wholesalers, persons authorized or entitled to supply medicines to the public (e.g., pharmacists and hospitals) and National Competent Authorities (NCAs). NCAs shall have access to the repositories system for supervising its functioning and investigating potential incidents of falsification, reimbursement, and pharmacovigilance or pharmaco-epidemiology.

### Data Contained in the European Medicines Verification System

**Figure 1:**
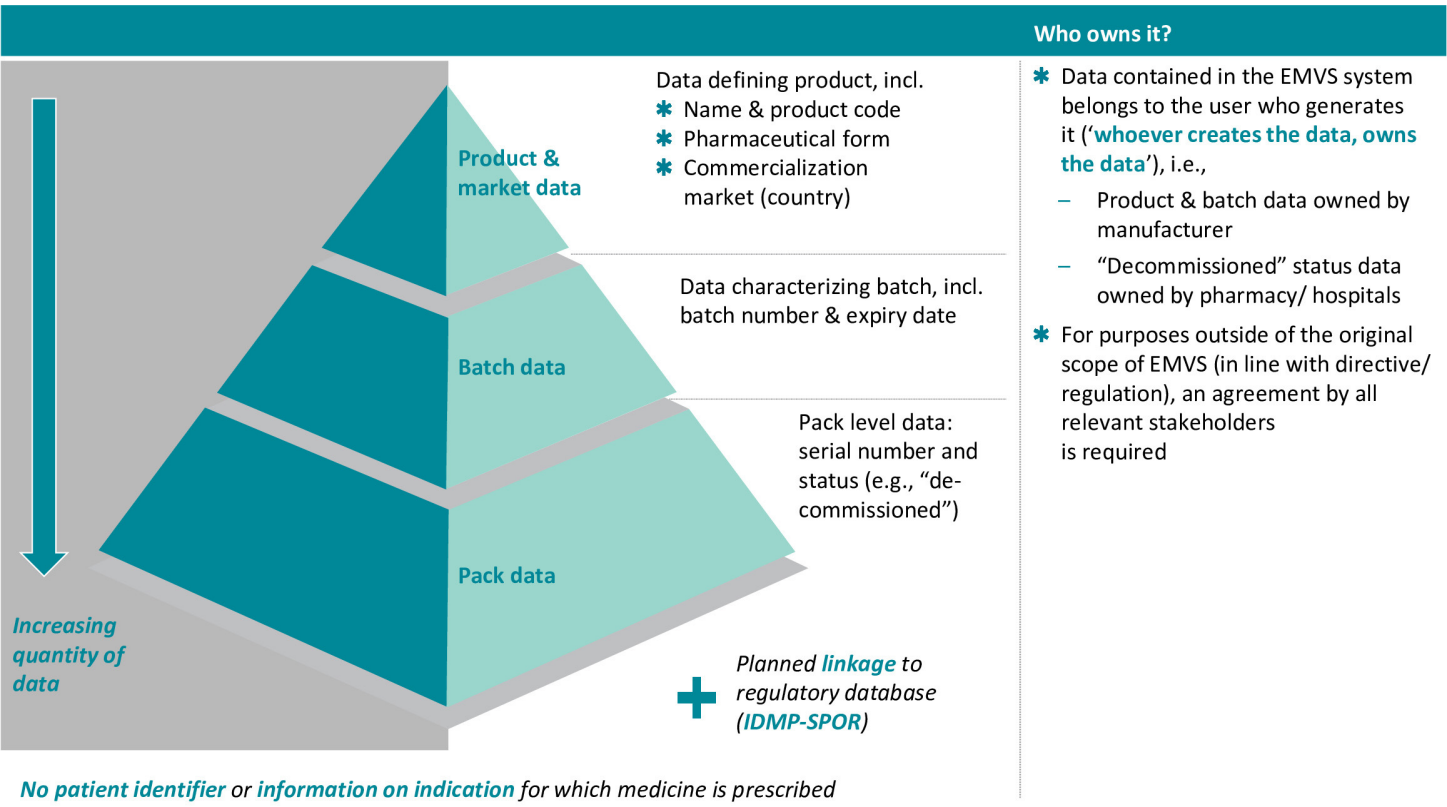
Overview of EMVS data contents and data ownership. Source: Internal EFPIA resource.

The EMVS holds the following information (see also Figure 1):

- Product Code (plus national reimbursement number if appropriate)
- Batch number
- Expiry date
- Serial number
- Product Master Data
- Marketing Authorisation Holder
- Manufacturer details (the assigned manufacturer of the batch)
- Current status of the unique serial number, i.e., active or de-commissioned—in case of “de-commissioned” also the detail, e.g., dispensed, recalled, stolen, etc.;
- By whom/where a change of status has happened
- Time and date of preceding changes.

Product Master Data includes the following

- Unique product code
- Product name and common name
- Pack type (which carries the serial number, e.g., bottle and carton)
- Form (e.g., tablet, capsules, and solution)
- Strength of the formulated drug (e.g., 20 mg and 1 mg/ml)
- Number of re-packable doses per pack (e.g., 10 tablets, capsules, etc.)
- List of contracted Wholesalers/3PL's (third party logistics).

The requirement of recording the status of the unique identifier (when decommissioned by persons authorized to dispense medicines to the public) is equivalent to the generation of sales data on a continued basis (this requirement for decommissioning also applies to parallel traders and to wholesalers under some circumstances).

### How Does the European Medicines Verification System Work in Practice?

Manufacturers place the safety features (a unique identifier/2D bar code and anti-tampering device) on each individual medicinal pack to be sold in the EU/EEA markets. Prior to batch release into a market or multiple markets, manufacturers upload the information contained in the Unique Identifier into the respective national repository system(s), part of the European Medicines Verification System.

For the purposes of the Falsified Medicines Directive, parallel traders are considered manufacturers and are therefore subject to the same requirements as original manufacturers. In the particular case of parallel traders, they are required to decommission the unique identifier of all packs from the source market and replace the safety features with equivalent features, including the upload of new unique identifiers in the repository system of the destination market (while recording a clear link at batch level between the two—this recording is also captured by the EU Hub).

Pharmacists are legally required to systematically verify—via the repository database(s)—the authenticity of each unique identifier (i.e., each pack), before dispensing it to the patient. Pharmacies will therefore only dispense a product if it is verified (i.e., information about the product is included in EMVS and all data elements of the unique identifier correspond to the correct information uploaded by the legitimate manufacturer). Upon dispensation, the unique identifier is “decommissioned” and its status within the system is listed as “decommissioned for dispense.”

**Figure 2:**
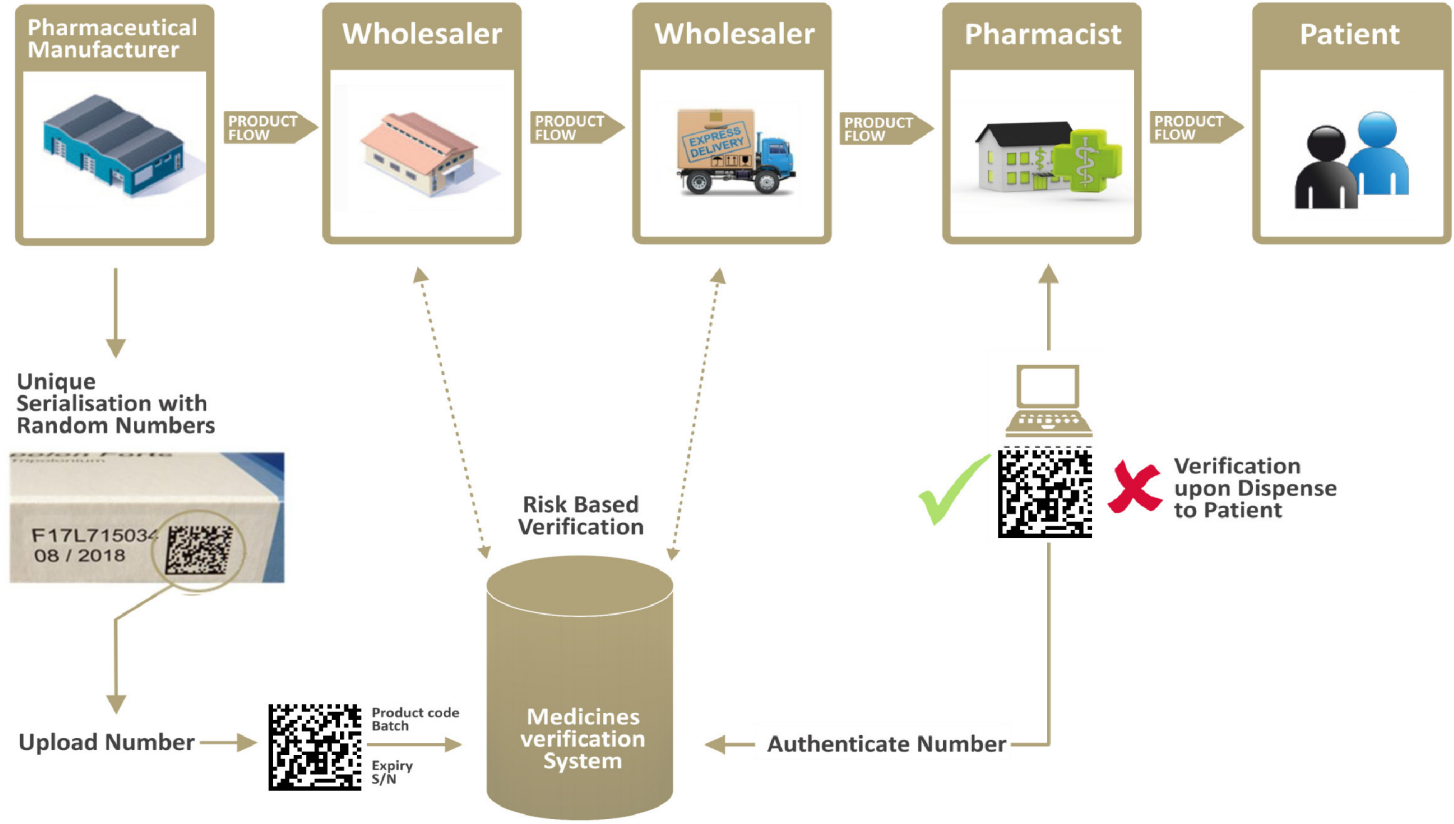
The verification flow along the supply chain. Source: Internal EFPIA resource.

On a risk-based approach, additional verification is being carried by wholesalers along the supply chain. For example, returns or products received from other wholesalers historically represented a higher risk of falsification; therefore, the legislation mandates wholesalers to systematically verify these two product categories. See Figure 2 for a visual representation of the supply chain and verification flow.

### Connection With EMA IDMP/SPOR Database and Additional Benefits to Be Derived

Article 33(e) of the Delegated Regulation requires that the code identifying the entry corresponding to the medicinal product bearing the unique identifier in the database referred to in Article 57 (1) (l) of Regulation (EC) N° 726/2004 be uploaded in the EMVS. Although the Delegated Regulation does not require de jure the linkage between EMVS and the future realization of the IDMP (SPOR) regulatory data repository^5^ (i.e., the database referred to in Article 57) this connection is de facto expected to take place. It is envisaged that MAHs will be able to upload partial master data (as opposed to a full data set) to the EMVS directly allowing the EMVS to “fetch” the regulatory approved master data values directly from SPOR. This is expected to lower the data exchange overhead for each MAH but also to improve the overall pan-European master data quality. The Delegated Regulation requires name and address details for the MAH, batch manufacturer and wholesalers. Whilst SPOR is out of scope for traders and wholesalers, a significant lowering of master data maintenance can be leveraged by allowing the master data to contain the SPOR Organization ID for MAH and manufacturers and linking to the organizational details within SPOR where the name and address information may be sought.

In addition, extra master data can be incorporated into the EMVS data set to improve the versatility and enhance potential up-stream use. The medicine classification code (e.g., ATC code) is not currently held by the EMVS as part of the master data but will be (mandatorily) available in the IDMP SPOR database and then uploaded in the EMVS once the IDMP/SPOR connection is realized.

Connection with SPOR is currently still a longer-term prospect as the EMA has yet to fully set-up the SPOR database itself.

### Data Access Into the System by Stakeholders

As a principle, the data contained in the EMVS system belongs to the user who generates this data when interacting with the EMVS (“whoever creates the data, owns the data”). The EMVS repositories system shall hold the following data components:

- Static data (i.e., the information listed under Article 33(2) of Commission Delegated Regulation (EU) No 161/2016)^6^ equivalent to manufacturers' uploaded data;
- Dynamic data i.e.:
  ° the status of the unique identifier, i.e., active or de-commissioned. In case of “de-commissioned” also the detail, e.g., dispensed, recalled, stolen, etc.;
  ° changes to the audit trail as referred to in Article 35(1), (g) of Commission Delegated Regulation (EU) No 161/2016, which contains a record of all operations concerning a unique identifier, of the users performing those operations and the nature of the operations.

As per the principle outlined above, dynamic data and static data contained in the EMVS system belong to the operator who generates the data when interacting with the system. This information must not be accessible for any other party, with exception of the static data and the information on the status of a unique identifier for the sole purpose of verification [Article 38(1) of Commission Delegated Regulation (EU) No 161/2016] and without prejudice to the right of access by national competent authorities as provided for under Article 39 of Commission Delegated Regulation (EU) No 161/2016.

### Data Access Into the System by Member States National Competent Authorities

Article 39(a) of the Delegated Regulation establishes that the legal entity establishing and managing a repository used to verify the authenticity of or to decommission the unique identifiers of medicinal products placed on the market in a Member State shall grant access to that repository and to the information contained therein, to competent authorities of that Member State for the purpose of supervising the functioning of the repositories and investigating potential incidents of falsification, reimbursement, pharmacovigilance, and pharmacoepidemiology. The Commission and NCAs have outlined the type of reports they are entitled and expect to receive from the European Medicines Verification System (the European hub^7^ and national repositories) for the purposes of investigations, reimbursement, pharmacovigilance, and pharmacoepidemiology. Specifically, a number of reports for Member States are to be generated outlining, for example, the number of product packs supplied, decommissioned and dispensed to patients in any given market/territory/post code, including the identity of all actors which have interacted (scanned or decommissioned) with any given pack. The system allows Member States direct access and possibility to extract data (through an Application Programming Interface and a Graphic User Interface as stipulated by Article 35(e) of the Delegated Regulation). Last but not least, additional reports are foreseen using medicinal classification such ATC codes (the addition of ATC codes would require an expansion of the master data in EMVS as detailed in the section above). Adding ATC codes to the EMVS master data would reduce the number of report requests made by the NCAs as it would allow the compilation of the required data in a single step rather than requesting multiple data sets (by product code) and combining the data subsequently.

## Discussion

Considering all of the above, we believe that the data stored in the interoperable network of national repositories being set up in the context of the Falsified Medicines Directive (Directive 2011/62/EU) and its Delegated Regulation 2016/161/EU on safety features can be used to provide additional intelligence to monitoring shortages.

This data could provide useful intelligence regarding the number of packs for all prescription products being supplied by manufacturers on the various EU markets, number of packs dispensed in national pharmacies, number of packs exported (and/or imported), as well as on the level of stocks present in the supply chain at country level. The real time information in the repositories can be analyzed according to very granular time frames (per day, per week, per month etc.) as well as per region (postal codes). That wealth of data would supplement information already provided by Marketing Authorisation Holders on manufacturing and quality related supply disruption to National Competent Authorities, and in providing information on causes and extent of shortages beyond manufacturing related issues, would greatly facilitate the detection and mitigation of genuine shortages.

In this respect, any National Competent Authority, by interrogating the data in the repository system on a Product level (by using the Product Code in the system) can identify the Net Stock Level of available medicinal packs in its territory at any given moment, by using the following formula:

$$\begin{array}{l} NS_{\left(n\right)}\;=\;{\left(U\right)}_{\left(m\right)}\;+\;U_{\left(pt\right)}-D_{\left(n\right)}-E_{\left(n\right)}-IMT_{\left(o\right)} \end{array}$$

Whereas:

NS_(n)_ = net stock level at national level

U_(m)_ = number of unique identifiers uploaded into the national market by the original manufacturer (corresponding to the number of physical packs released on the market)

U(_pt)_ = number of unique identifiers uploaded into the national market by parallel trader(s) (corresponding to the number of physical packs imported in the national market)

D_(n)_ = number of unique identifiers decommissioned at national level by persons authorized or entitled to supply medicinal products to the public, typically community pharmacists, hospital pharmacists or in some cases wholesalers (on behalf of Article 23 actors) (corresponding to the number of physical packs which have been dispensed to patients within the territory)

E_(n)_ = number of unique identifiers decommissioned for export at national level by parallel traders (corresponding to the number of physical packs exported from the national market)

IMT_(o)_ = number of unique identifiers destined for the national market but decommissioned in other markets via the Inter Market Functionality^8^ of the system.

The present formula can be enlarged depending on the needs of the National Competent Authorities as D_(n)_ can be further disentangled to provide an overview of decommissioned activities per sub-category/reason (there are seven possible sub-categories/reasons: “dispensed,” “exported from EU,” “destroyed,” “stolen,” “sample for NCA,” “clinical trial investigative material,” or “locked”).

Moreover, as the National Competent Authorities have access to the data contained in the system via an Application Programing Interface which enables them to generate pre-defined reports, such reports can be generated on any time basis (monthly, weekly, daily, hourly, etc.) thus enabling the National Competent Authorities to monitor the evolution through time of the net stock level in their respective territory.

Several confounding factors can complicate the use of the formula by National Competent Authorities and we outline them below:

### Confounding Factor 1: Data Completion

The Falsified Medicines Directive and its Delegated Regulation currently apply only for prescription medicines. Therefore, the data contained in the repository systems can only be used to provide further intelligence to monitor shortages of prescription-only medicines (medicines in scope of the Falsified Medicines Directive).

The Delegated Regulation went into force on 9 February 2019 and all corresponding obligations on supply chain actors started to apply only for products released for sale on EU/EEA markets as of 9 February 2019. All prescription medicinal packs released for sale, un-serialized, prior to that date were grandfathered into the legislation and continue to circulate in the supply chain in parallel with serialized packs until they are dispensed to patients or they expire. Considering 5 years as the typical expiry date of a medicinal pack the system will contain full data for all corresponding prescription medicinal packs on the market only as of 9 February 2024. However, with each passing day, the data in the system gets sharper and sharper and more complete as more serialized medicinal packs are released on the market and more un-serialized packs (pre-9 February 2019) are either dispensed or expire. Furthermore, nearly 2 years and a half after the entry into force of the Delegated Regulation, we can consider that most if not all highly demanded products have only serialized packs circulating on the market at this stage.

### Confounding Factor 2: Issue of Multimarket Packs

Some medicinal products are released for sale in multiple markets at the same time (and are packaged according to the packaging requirements—for example the language of the patient information leaflet—of multiple countries). In such cases, a manufacturer will upload the unique identifiers of the batch released for sale into the repository systems of the countries in the multi-market pack cluster. When a medicinal pack is decommissioned for dispense in one market then the unique identifier is automatically decommissioned in all markets.

As such, the application of the net stock level formula outlined above is complicated by the existence of decommissioning data in multiple national systems. As such, there is a need for collaboration and data exchange between the National Competent Authorities in the respective markets of the cluster so that the data can be interrogated and aggregated for all markets of the cluster. The formula outlined above still holds, but will show the net stock level across the markets in the cluster.

### Confounding Factor 3: Issue of Multi-Source Products

Currently the data in the system can only be identified according to Product Code (corresponding to a physical Stock Keeping Unit). While this is straightforward for single source products (typically on-patent medicines) the analysis is further complicated in the case of multi-source products (typically off-patent medicines) as National Competent Authorities need to analyze the net stock level in parallel for multiple medicinal products which are deemed interchangeable for the treatment of a specific condition in order to identify the risk of shortage. Currently this can be done in a manual way by running and aggregating multiple reports, however, once the connection with the EMA IDMP/SPOR database National Competent Authorities will have the possibility to run reports on an ATC class level. Such reports would automatically assembly the required data for multiple products in a specific class.

## Recommendation: Proposal for Collaboration Between National Competent Authorities and Manufacturers in Order to Proactively Monitor Country Stock Levels

**Figure 3:**
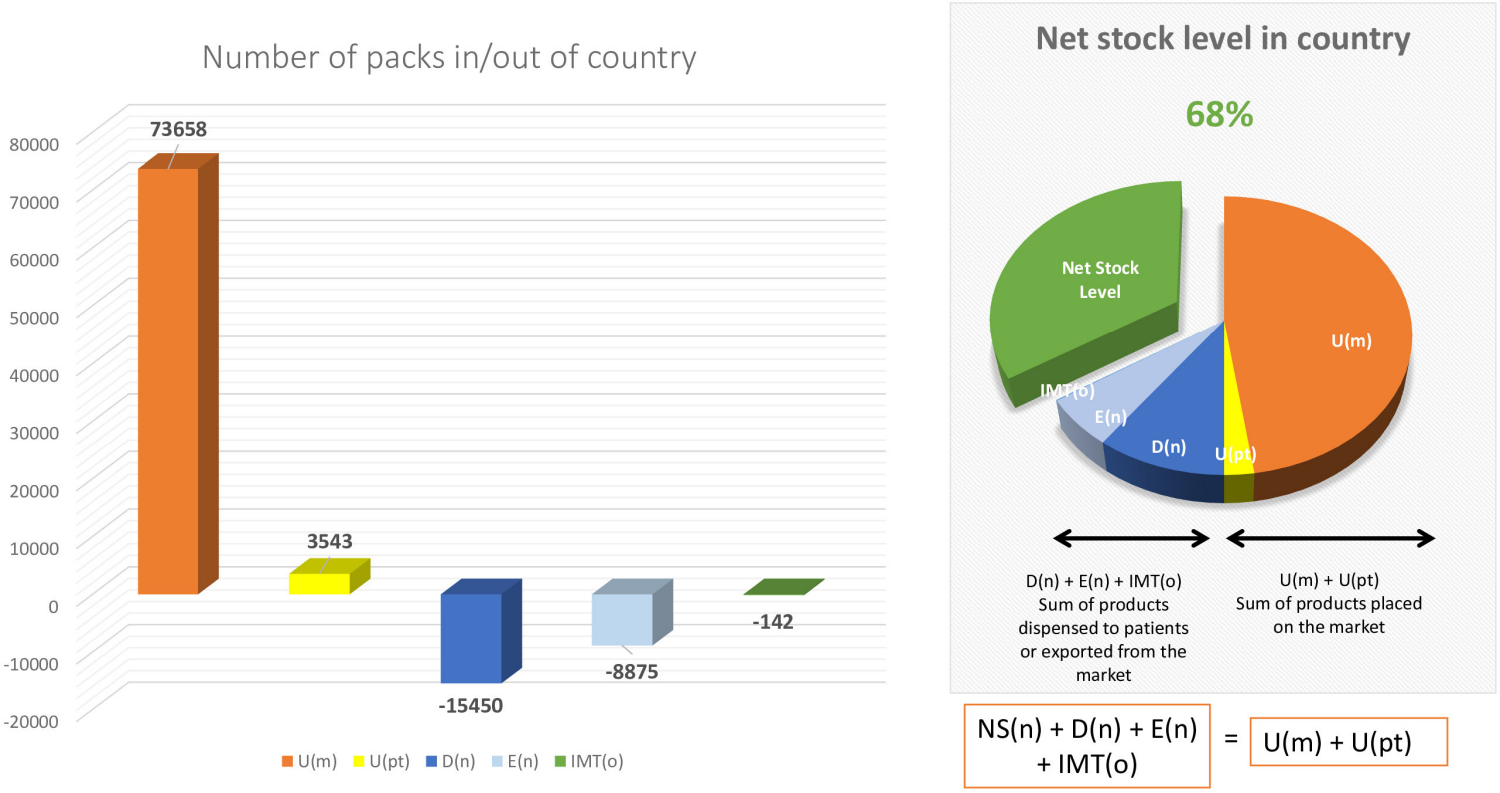
Example of detailed dashboard. Source: Authors own proposal for a detailed dashboard.

Continuously monitoring the Net Stock Level of a medicinal product in a Member State allows for proactive measures to be taken both by National Competent Authorities and by manufacturers to avoid the national Net Stock Level to fall in negative territory. For this purpose, we propose the creation of a “dashboard” type early-warning mechanism (see Figure 3 for an example of such dashboard) whereas a National Competent Authority would notify the manufacturers once a certain minimum threshold is reached at national level (for example, Net Stock Level reaches 20%) so that the manufacturer can take actions to pre-emptively re-supply the respective market. As the data access rules within the system are strict, this early warning notification system can only be triggered by National Competent Authorities (as they have access to the full data) and be exchanged only at aggregate level. Depending on the type of product different thresholds can be set for different medicines, in collaboration with the original manufacturer (depending on manufacturing lead time, availability of stock in other markets, etc.).

Such a theoretical proposal would require further analysis and potentially the set-up of a pilot initiative in a (or multiple) Member State(s) with the involvement of the National Competent Authorities. Such a pilot initiative would allow for the testing of the above-mentioned formula as well as the proposed dashboard and gather additional intelligence from real-life use and experiences. Several practical questions could be answered in such a pilot setting, for example: how often do Member States need to consult the data in the repository systems, are there any data gaps (now and in the future) in the system, does the data in the system reflect the reality on the ground with sufficient accuracy to make it actionable, are there any further considerations needed to be taken into account depending on the type of products or territory, how can the collaboration between multiple Member States work in practice, what are the various minimum thresholds for the dashboard etc.

### Enforcement of Public Service Obligations on Supply Chain Actors

Last but not least, the National Competent Authority may further use the intelligence derived from continuously monitoring the net stock levels to consider taking measures to prevent or address shortages, such as restricting the free movement of goods within the EU, for example when the Net Stock Level indicated by the dashboard descends under a predefined level. In line with Article 36 of the Treaty on the Functioning of the European Union (TFEU) Member States are entitled to take measures to ensure security of supply where there is a genuine risk of shortages. Such measures include mandatory pre-export notification, consent requirements imposed on wholesalers, and restrictions on pharmacist wholesale activities. In exceptional circumstances, the imposition of limited export bans may be justified in respect of those medicines where there is a demonstrated supply shortage and consequent risk to patient safety.

Such measures are lawful under EU law free movement principles provided they are taken in response to a genuine public health risk and are proportionate in that they are appropriate to achieve the stated public health objective, and are not more restrictive than is necessary to achieve their legitimate objective^9^.

Even without such drastic measures, rigorous enforcement of public service obligations not only of MAHs but also of other actors in the distribution chain could help to reduce shortages especially in lower income Member States (see, for example, the measures introduced into the Polish Pharmaceutical Law in 2015 and 2019).

Regulatory obligations applicable to Marketing Authorization holders (MAHs) are embodied in two main provisions of the EU legislation relating to the monitoring of supply and reporting of shortages:

- MAHs' obligation, in case a “*product ceases to be placed on the market of a Member State, either temporarily or permanently*” to “*notify the competent authority of that Member State…no less than two months before the interruption in the placing on the market of the product*,”^10^ and
- MAHs' and distributors' public service obligations to ensure “*within the limits of their responsibilities, appropriate and continued supplies of medicinal product[s] to pharmacies and persons authorized to supply medicinal products so that* *the* ***needs of patients****in the Member State in question are covered*” (5).

Marketing authorization holders (“MAHs”) as well as wholesalers have a fundamental “public service” obligation to ensure “*within the limits of their responsibilities, appropriate and continued supplies of medicinal product[s] to pharmacies and persons authorized to supply medicinal products so that the needs of patients in the Member State in question are covered*” (5). Member States should evaluate the limits of the responsibilities of MAHs and wholesale distributors on a product-by-product basis. There are scenarios where the MAH is not responsible for the shortage. For example, when the MAH's supply meets ordinary orders, in real-time, in relation to demand from patients of the Member State concerned, but a shortage is caused by a distributor's export/supply of medicines to another customer in a different Member State, without the MAH being aware. Sometimes, this takes place in spite of a manufacturer introduced quota for supplying the market based on expected patient needs. Another example is shortages caused by increased demand due to a shortage in the Member State of an alternative medicinal product produced by another company^11^.

The above-mentioned analysis can be done using the data from the repository system on a case-by-case basis.

Public service obligations imposed on MAHs should not lead to an obligation to sell to all entities with a wholesale license in a market nor to an obligation to fulfill orders in full on a first-come, first-served basis. This would result in competitive distortions in the wholesaler market and increase the risk of the MAH being unable to supply other wholesalers in accordance with their usual orders, hence jeopardizing that all pharmacies/patients' demand is met. The MAH should at all times be allowed to manage its supply chain in a way that best ensures that patient needs are met which is the fundamental obligation of the authorization holder (see Art. 81 Directive 2001/83). The public service obligations of other actors in the supply chain should be enforced by national authorities and should not be the responsibility of MAHs.

## Author Contributions

MR and FB designed the manuscript. MR compiled the first draft and subsequent iterations of the article and FB revised the manuscript. Both authors read, commented on, contributed to the manuscript for the accuracy of the content, finally approved the version to be published, and agreed to be accountable for all aspects of the work in terms of its accuracy and integrity.

## Conflict of Interest

FB and MR are currently employed full time by the European Federation of Pharmaceutical Industries and Associations (EFPIA), representing research-based pharmaceutical manufacturers in Europe.

## Footnotes

^1^EFPIA—together with other EU Health stakeholders—have called upon the Commission to set up a High-Level Forum on Better Access to Healthcare Innovation involving Member States, Commission, and stakeholders to discuss drivers leading to unequal availability across Europe. Decisions by economic operators to bring a medicinal product on to the market are always driven and influenced by a combination of factors (regulatory, legal, and commercial). Thus, a holistic approach must be used to find solutions aimed at increasing availability. Eliminating one barrier will not significantly change the situation or lead to any satisfactory improvements in availability.

^2^See comments by EFPIA, Medicines for Europe and EASGP on EMA/HMA Guidance on detection and notification of shortages of medicinal products for Marketing Authorization Holders in the Union: the definition included in the EMA/HMA Guidance refers to national demand rather than patients needs and therefore implies that it is the supply chain (e.g., wholesalers and parallel traders) that defines the existence of manufacturers' shortages irrespective of patient needs. The EMA/HMA definition goes beyond the responsibilities of a marketing authorization holder and the scope as identified in Article 81 of Directive 2001/83/EU.

^3^See briefing from European Parliamentary Research Service, Nicole Scholz, April 2020, European Parliament, available at: https://www.europarl.europa.eu/RegData/etudes/BRIE/2020/649402/EPRS_BRI(2020)649402_EN.pdf.

^4^Not all products have the same criticality and impact with regards to patient needs and supply. The fact that a medicine is not available in a specific presentation but is available in other presentations cannot be put on the same footing with a shortage of a life supporting or life sustaining medicine.

^5^The European Medicines Agency (EMA) is implementing the ISO IDMP standards for the identification of medicinal products in a phased programme, based on the four domains of master data in pharmaceutical regulatory processes: Substance: harmonized data and definitions to uniquely identify the ingredients and materials that constitute a medicinal product; Product: harmonized data and definitions to uniquely identify a medicinal product based on regulated information (e.g., marketing authorization, packaging, and medicinal information); Organization: data comprising organization name and location address, for organizations, such as marketing authorization holders, sponsors, regulatory authorities, and manufacturers; Referential (SPOR) data: lists of terms (controlled vocabularies) to describe attributes of products, e.g., lists of dosage forms, units of measurement, and routes of administration.

^6^These data are: (a) the data elements of the unique identifier, (b) the coding scheme of the product code, (c) the name, form, strength, and pack size of the product, (d) the Member State(s) where the product is intended to be placed on the market, (e) any corresponding entry relating to the product in the database of medicinal products accessible to the public referred to in Article 57(1)(l) of Regulation 726/2004 on the autorisation and supervision of medicinal products, (f) the identity of the manufacturer placing the safety features, (g) the identity of the marketing authorization holder, and (h) the latter's list of designated wholesalers.

^7^The European hub is considered as a repository and will be supervised by the national competent authority of the Member State where it is located. That Member State (and by extension all Member States) is entitled to access and use the data contained in the hub.

^8^An Inter Market Functionality is triggered when a medicinal product destined for market X has been decommissioned for dispense in market Y, without having been parallel traded (such examples include: name patient basis prescriptions, Art 126a products or others).

^9^See for example Case C-169/07 *Hartlauer v Wiener Landesregierung*, ECLI:EU:C:2009:141, para. 55.

^10^Article 23a of Directive 2001/83/EC. See also Article 123 for MAHs' notification obligations in case of suspension or withdrawal of the marketing of products, or the withdrawal or failure to renew the marketing authorization for products.

^11^Paper on the obligation of continuous supply to tackle the problem of shortages of medicines agreed by the *ad-hoc* technical meeting under the Pharmaceutical Committee on shortages of medicines on 25 May 2018, European Commission.
